# Congenital cystic adenomatoid malformation in adults detected after infection

**DOI:** 10.1002/rcr2.364

**Published:** 2018-09-14

**Authors:** Hiroyuki Kagawa, Keisuke Miki, Mari Miki, Koji Urasaki, Seigo Kitada

**Affiliations:** ^1^ Department of Respiratory Medicine National Hospital Organization Toneyama National Hospital Toyonaka Japan; ^2^ Department of Laboratory Medicine and Pathology National Hospital Organization Toneyama National Hospital Toyonaka Japan

**Keywords:** Adults, congenital cystic adenomatoid malformation, nontuberculous mycobacterial infection

## Abstract

Congenital cystic adenomatoid malformation (CCAM) is a benign congenital tumour in which a part of the lung becomes polycystic. Case 1 was a 64‐year‐old male who was diagnosed with pneumonia, with multiple cysts in the right lower lung lobe, using chest computed tomography (CT). After treatment of the pneumonia, including *Mycobacterium abscessus*, a right lower lobectomy was performed. Case 2 was a 41‐year‐old male who had suffered from pneumonia many times since his youth. Polycystic and infiltrative shadows were observed on chest CT. After treatment of the pneumonia, a right lower lobectomy was performed. Pathologically, both the cases were diagnosed as CCAM type 1. Although CCAM in adults is very rare, it should be considered in the differential diagnosis of cases with repeated pneumonia due to suspected congenital cystic disease. CCAM is better detectable with chest CT and requires active surgical treatment.

## Introduction

Congenital cystic adenomatoid malformation (CCAM) is a congenital disease in which adenomatous hyperplasia of the bronchiolar epithelium leads to the formation of numerous cysts 1. In this report, we describe two adult patients in whom pulmonary lobectomies were performed following pneumonia, which confirmed the diagnosis of CCAM.

## Case Report

### Case 1

**Figure 1:**
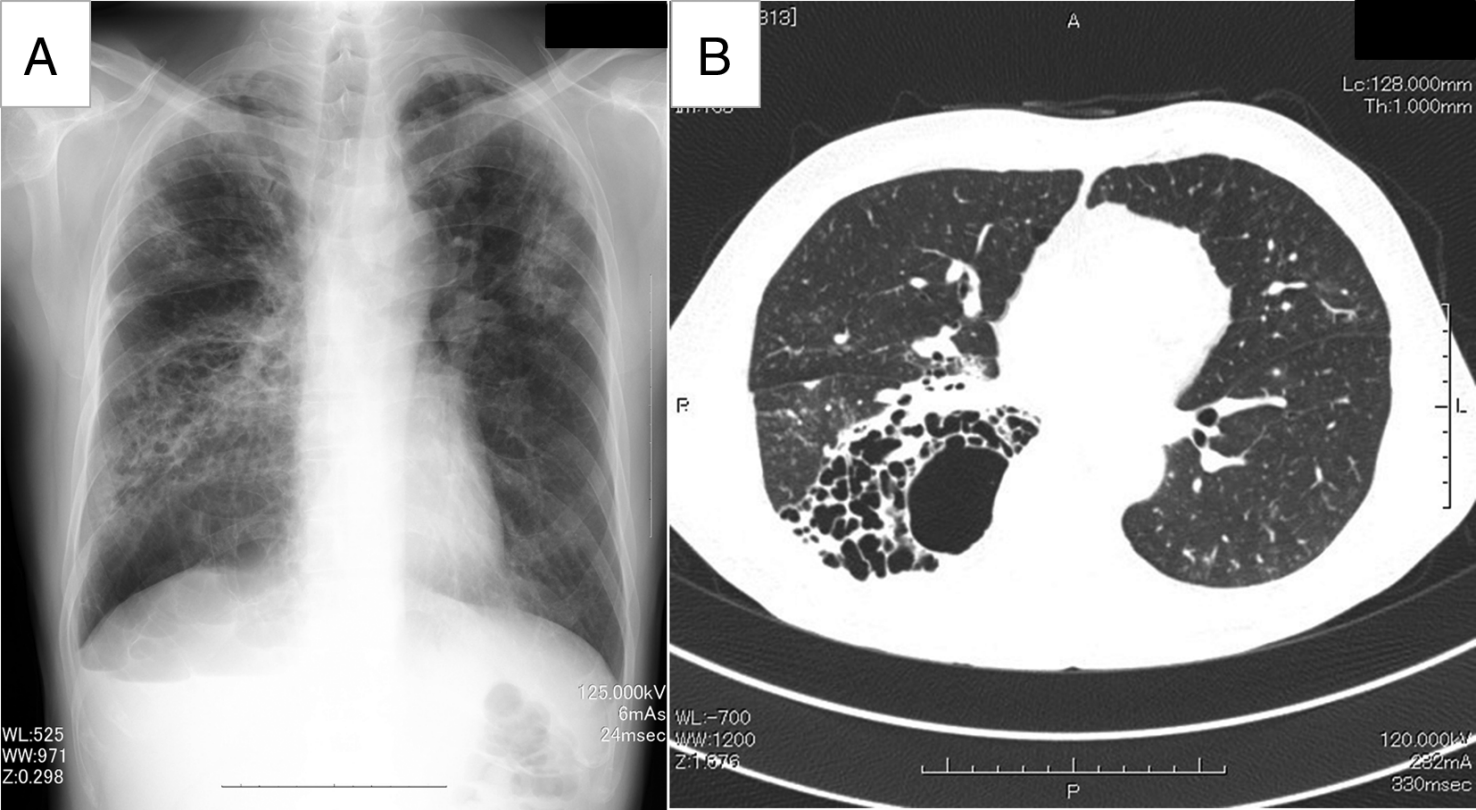
Case 1: (A) chest X‐ray image at admission showed infiltrative shadows in the left upper and right lower lung fields. (B) Chest computed tomography (CT) scan at admission showed multilocular cystic shadows together with air fluid levels in the right lower lobe and patchy shadows and cord‐like shadows in both the upper lung lobes.

A 64‐year‐old man presented with a fever and was diagnosed with pneumonia. He had a history of recurrent pneumonia. Chest computed tomography (CT) showed numerous cysts in the right lower lobe in addition to infiltrative shadows. *Mycobacterium abscessus* was identified in the sputum. Chest X‐ray images showed infiltrative shadows in the left upper and right lower lung fields (Fig. 1A). Chest CT showed multilocular cystic shadows (Fig. 1B). After treating the pneumonia, an open right lower lobectomy was performed. Histopathological evaluation of the surgical specimen indicated polycystic lesions in the lower lobe of the right lung. There was a dark brown pus‐like liquid in the lumen, but bacterial culture was negative. The large cystic lesions had smaller cysts scattered around them. The inner surface of the cysts was lined with ciliated bronchial epithelium that was not atypical, and there were aggregates of small lymphocytes in the surrounding stroma. The cyst wall was covered with ciliated columnar epithelium.

### Case 2

**Figure 2:**
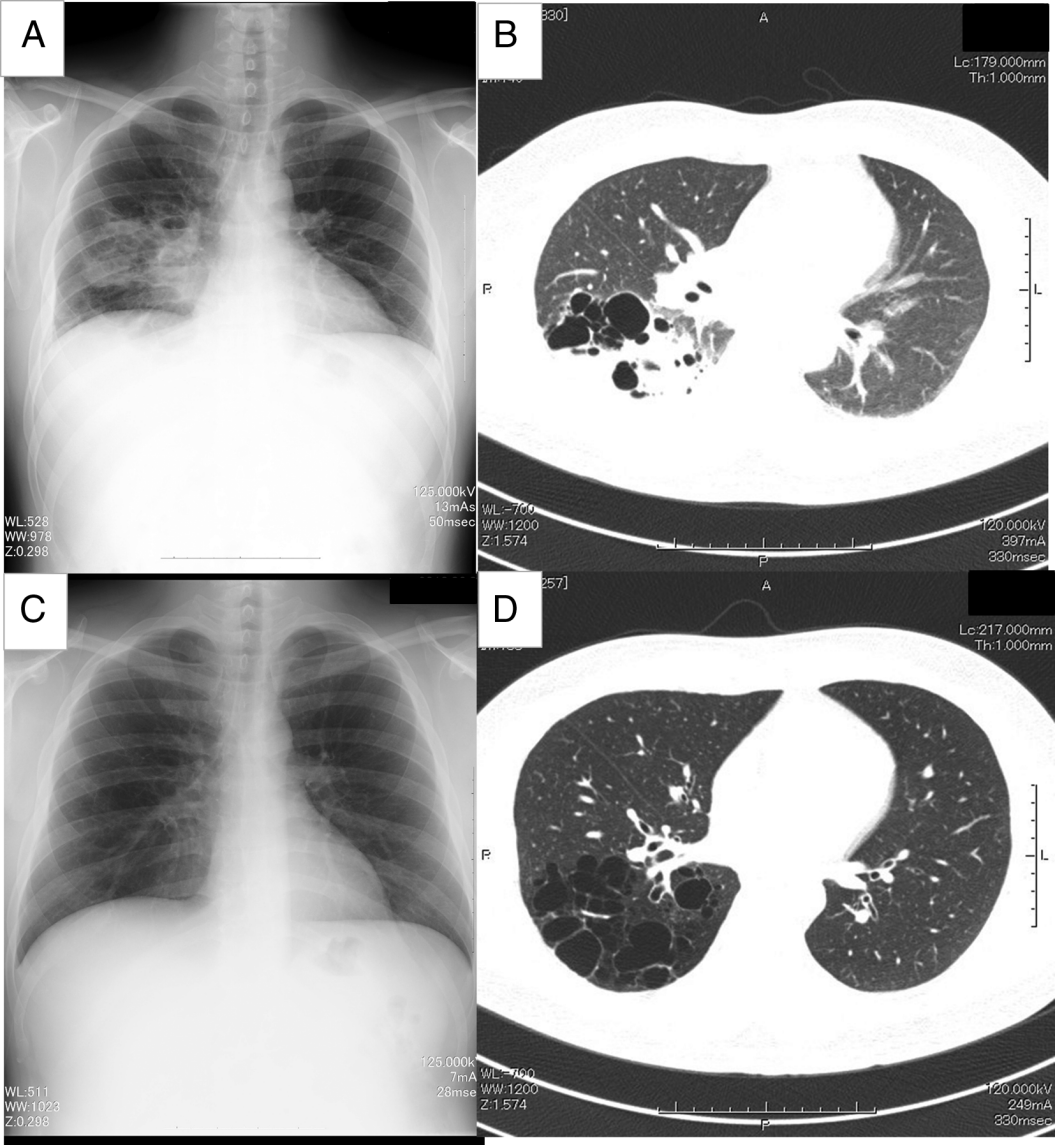
Case 2: (A) chest X‐ray at admission showed infiltrative shadows in the right lower lung field. (B) Chest CT scan at admission showed numerous cystic lesions and infiltrative shadows in the right lower lobe. After treatment for pneumonia, there was improvement in the above shadows on both (C) chest X‐ray and (D) chest CT scan.

A 41‐year‐old man was diagnosed with pneumonia. His past medical history included recurrent pneumonia. Chest X‐ray showed infiltrative shadows in the right lower lung field (Fig. 2A). Chest CT showed numerous cystic lesions and infiltrative shadows in the right lower lobe (Fig. 2B). An open right lower lobectomy was performed three months after treating the pneumonia. Histopathological evaluation of the surgical specimen revealed numerous cysts of up to 4 cm in diameter in the lungs.

**Figure 3:**
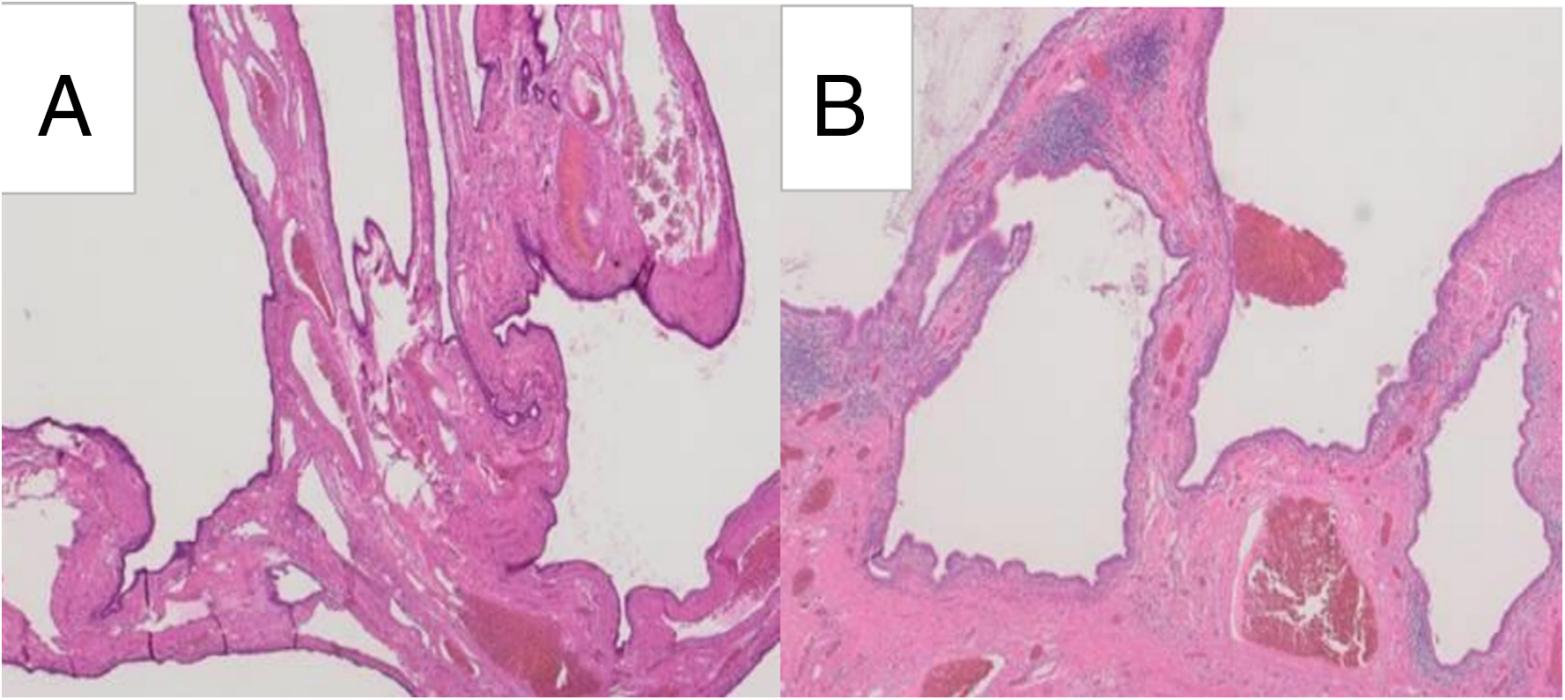
Histological findings of the resected lung (Hematoxylin‐Eosin staining, 20×). In both cases, histological findings of the resected specimen showed multiple cysts that were composed of fibrous walls lined by ciliated columnar cells, with no bronchial cartilage (Case1: A, Case2: B).

In both cases, histological findings of the resected specimen showed multiple cysts that were composed of fibrous walls lined by ciliated columnar cells, with no bronchial cartilage (Fig. 3A and 3B). Therefore, the pathologies were diagnosed as CCAM type 1. Atypical cells were not identified.

## Discussion

The detection of CCAM in adulthood, after the age of 18 years, is extremely rare, and only 14 such cases (10 males and 4 females) have been reported in Japan, including our cases. In these cases, the average age at onset was 46.5 years. In nine cases, CCAM was detected following symptoms such as fever, cough, and sputum, which were mainly associated with an infection, while the other five cases were asymptomatic, and CCAM was detected during an unrelated medical examination. The right lobe was affected in nine cases, while the left lobe was affected in five cases. Most of the cases were type 1 2 CCAM (10 of 14 cases). Although a biopsy helps to confirm the diagnosis of CCAM, other diseases that must be distinguished from this disease include pulmonary sequestration, bronchial atresia, bronchogenic cysts, and bronchiectasis. In both cases in this study, abnormal blood vessels were not found on preoperative contrast‐enhanced CT, and abnormal blood flow suggesting pulmonary sequestration was also not found in the surgical findings. Both the cases were diagnosed as negative for the above‐mentioned diseases as the pathological findings did not indicate bronchial disruption or inflammatory destruction of the bronchi, and there was an absence of cartilage in the cyst wall. Although CCAM is a congenital disease, the abnormalities in our patients were not previously detected because the lesions did not appear on chest X‐ray, and they only increased with repeated inflammation, reaching a stage where they could be detected. Even if health‐care providers were able to identify the abnormality, the entire pathological picture could not be understood due to insufficient information, and hence, the patients were only treated for pneumonia or their symptoms and not for CCAM, and the need for surgery was not considered because the patients were subsequently routinely followed up, were young, or had comparatively mild symptoms. Particularly in Case 2, identification of multiple cysts on chest X‐ray images after improvement of pneumonia was difficult, and this is believed to be the reason for the abnormality not being identified on chest X‐rays before the patient’s current presentation (Fig. 2C). Chest CT (Fig. 2D) might be a better diagnostic modality than X‐rays for cystic lung diseases like CCAM, as previously reported 3. In adults, modifications to the primary disease due to secondary changes and infections also occur, as in Case 1, making it clinically difficult to distinguish congenital abnormalities from similar lesions that have developed later. Fourteen cases of adult‐onset CCAM have been reported in Japan, including our cases. However, none of the cases had *M. abscessus* infection as a complication, which makes our case with non‐tuberculous mycobacteria (NTM) interesting.

Because the risk of recurrence of infection in the cysts of CCAM is high, surgery is the main treatment in these patients. Furthermore, adenocarcinoma as a complication, as has been previously reported 4, can occur due to the progressive growth of mucus cells accompanying metaplasia arising from the cyst wall and their subsequent development into adenocarcinoma. Moncrieff et al. 5 reported that reoperation was necessary in cases where only the cysts were excised and that cystic lesions were found in the remaining lung lobes after partial resection. Because adenocarcinoma may remain due to the persistence of the cyst wall after treatment of the pneumonia, surgical treatment by lung lobectomy is recommended, taking care to ensure that an adequate surgical margin is maintained. Surgery should be performed for CCAM even if the patient is asymptomatic.

Our cases highlight the fact that, in patients with pneumonia recurring at the same location, medical professionals should keep in mind the possibility of congenital cystic diseases, such as CCAM, as a differential diagnosis. Chest CT is preferable to chest X‐ray in the diagnosis of CCAM. Further evaluation of more cases is necessary.

## Disclosure statement

Appropriate written informed consent was obtained from the patients for publication of these case reports and accompanying images.
